# Anatomico-physiological Notes on the Structure of the Heart of the Sturgeon and of the Ray

**Published:** 1848-12

**Authors:** 


*Anatomico-physiological Notes on the Structure of the Heart of the Sturgeon and of the Ray.* By M. Parchappe. *Translated from the "Comptes Rendus" of July*, 1848. By James Bryan, M. D., Professor of Surgery in Geneva Medical College, President of the Philadelphia College of Physicians and Surgeons, &c.

I. *The anatomico-physiological signification of the peculiar structure of the heart of the sturgeon.*—Valsalva, cited by Morgani, advanced the idea that the heart of the sturgeon contains glands which throw out a dark fluid into the ventricle. Huhl, Hoelrenter, Baer, and other anatomists, have described, on the surface of the ventricle of the arterial bulb, and even on the auricle of the heart of the sturgeon, numerous and voluminous enlargements like lobes, having cavities, which did not communicate with the bulb or ventricle.

I have established, by my researches, that these lobulated projections on the heart of the sturgeon, represent the free external surface of sanguine cells, which adhere to the external surface of the ventricle and bulb; that these blood cells, which have no communication with the cavities of the heart, communicate with each other, and are a continuation of the arterial and venous vessels of the heart; that the walls of these cells are muscular; that the venous and arterial vessels whose origin or termination begins with these cells, leave the heart on a level with the circular ridge, to cross the pericardium, and unite with the other side of this envelope of the venous and arterial trunks, and form in the sturgeon, as I have described in the eel, the basis of cords, formerly considered as simple ligaments.

I interpret the physiology of these anatomical arrangements in the following manner:

1. That the contractile blood-cells of the surface of the heart of the sturgeon act like the ordinary cavities of the heart, the double function of a reservoir, and a propelling agent to the blood.

2. That these cells partake of the characters of the auricle, in receiving blood by the vessels, and of the ventricle in propelling blood through the vessels.

3. That these cells are destined as reservoirs, to receive the blood from the coronary arteries, vivified by the branchial respiration, as agents of propulsion, to drive this vivified blood through the coronary veins to the venous sinus.

4. That the cardiac veins and arteries, do not constitute exclusively, in the heart of the sturgeon, the ordinary and simple circle, at least of the arterial blood, after having traversed a capillary system for the purposes of nutrition, returning disarterialised to the auricle of the heart; that they form canals as it were of another circle, in which the contractile cells occupy the position of capillary vessels, a circle by means of which a considerable portion of the blood arterialised by branchial respiration is turned by the arteries into the blood-cells, from whence by the contraction of these cells, it is driven through the veins to the sinus, which carries to the auricle the venous blood of the system.

II. *Peculiar conformation of the venous sinus of the pericardium, in the heart of the ringed ray.*—Monro first, then other anatomists, have admitted as a remarkable peculiarity, the communication of the pericardium with the abdominal cavity in the ray, and in several other cartilaginous fishes. I am convinced that the cavity of the pericardium in the ringed ray, is formed by a blind pouch as in the higher animals; and I have observed in this fish a conformation of the pericardium, and an arrangement of the venous sinus, very different from the usual descriptions given by authors.

The venous sinus which opens directly into the auricle of the ray, is situated within the pericardium. It is formed by the union at an acute angle of two considerable vessels, which descend along and adhere to the posterior wall of the ventricle. Arrived at the inferior margin of the ventricle, they remain united by a membrane, which is attached between them, to the ventricle, and accompanies them to the point where they go out of the pericardium, that is to say, to the right and left angles of the base of this cavity, supported by the diaphragm. From this arrangement it follows that the cavity of the pericardium, in its inferior half, is divided from above downwards into two compartments, the one anterior and the other posterior, like a triangular partition. The membrane between the vessels forms the base of the triangle adhering to the pericardium ; the two vessels form the free sides, in the cavity of the pericardium : the ventricle completes above and in the middle, this partition, whose summit corresponds to the venous sinus.

The right and left pouches of the outside, placed like a bag above the base of the ventricle, behind the arterial bulb, are supported on this inferior extremity, on each side of the ventricle on the anterior surface of the partition. Behind the partition is the back compartment, which communicates over the venous vessels with the anterior compartment, and terminates below in a *cul de sac*, which rests upon the diaphragm.

The union of the venous vessels, in the cavity of the pericardium behind the ventricle, forms a horizontal sinus, which opens into the auricle, and which may be called *superior and median sinus.* These two vessels open below the diaphragm, and outside of the pericardium in two larger sinuses, which may be called *inferior and lateral sinuses.* These two sinuses are exactly symmetrical. The venous canal arises from the superior part of each, by a round opening. Each of them, besides, communicates by its external part, with a vein imbedded in the cartilaginous mass of the branchial arches; by its inferior portion with the corresponding hepatic vein.

Both the hepatic veins, before opening into the sinuses, are considerably enlarged, and form two ovoid pouches, joined to the diaphragm and to the inferior wall of the sinus. The communication of each of these pouches with the cavity of the corresponding sinus, is made by a round opening, hardly two millimetres in diameter, pierced in the membranous partition of the sinus and of the pouch, and presenting on its arch a species of pad, from which fasciculi of white fibres radiate.

III. *Active valvular arrangement of the auricular opening of the venous sinus in the ray.*—The opening of the superior and middle venous sinus is found in the auricle of the ray, above and behind the auriculo-ventricular orifice, at the union of the two pouches of the auricular cavity. The orifice of the venous sinus is transformed into a long slit, extended from the anterior to the posterior wall of the auricle, by two semi-lunar valves. The superior valve forms the superior lip of the opening, placed on the base of the ventricle, and extends by one of its horns over the posterior wall; by the other and smaller, over the anterior wall. The inferior valve forms the inferior lip of the opening, and extends, in a parallel direction so as, by its horns, to reach the extremity of the horns of the superior valve, and to form a commissure on each side.

Both angles or commissures of the valvular slit close upon thick muscular facicles, from which smaller muscular fibres radiate, to cover the anterior and posterior walls of the auricle. Each of these valvules have in their structure muscular fibres, dispersed length-wise.

We cannot but observe, in studying the whole of this delicate structure, that the orifice of the venous sinus is actively closed by the contraction of muscular fibres, at the instant of the systole of the auricle, like a button-hole whose lips are retracted, at the same time that a double force draws its angles in opposite directions.

